# Implant migration and clinical outcomes with cemented vs cementless Motec cups in total trapeziometacarpal arthroplasty: a 10-year prospective RSA cohort study of 69 patients

**DOI:** 10.2340/17453674.2026.46544

**Published:** 2026-09-09

**Authors:** Emil RAUTIO, Maiken STILLING, Torben B HANSEN, Janni K THILLEMANN

**Affiliations:** 1 Department of Orthopedics, University Clinic for Hand, Hip and Knee Surgery, Gødstrup Hospital, Herning; 2 Department of Clinical Medicine, Aarhus University, Aarhus N; 3 AutoRSA Research Group, Orthopedic Research Unit, Aarhus University Hospital, Aarhus N; 4 Department of Orthopedic Surgery, Aarhus University Hospital, Aarhus N, Denmark

## Abstract

**Background and purpose:**

Few clinical studies have assessed migration of trapeziometacarpal implants, and long-term outcomes are warranted. We aimed to evaluate 10-year migration, clinical outcomes, and revisions of cementless and cemented Motec cups. Additionally, early migration of revised and non-revised cups, and the association between clinical outcomes and cup revision were investigated.

**Methods:**

Radiostereometric analysis (RSA) was performed after cast removal (baseline = 3 weeks), at 3 months, 1, 2, 5, and 10 years. Clinical outcomes and revisions were evaluated prospectively.

**Results:**

22 cementless Motec titanium-screw cups were inserted from 2009 to 2010, followed by 47 cemented Motec all-polyethylene cups inserted with Palacos bone cement from 2011 to 2012. At the 10-year follow-up, mean cup total translation (TT) was 0.79 mm (standard error [SE] 0.55) for cementless cups (n = 3) and 1.33 mm (SE 0.30) for cemented cups (n = 10), with cemented cups demonstrating 0.54 mm (CI –0.68 to 1.77) higher translation. 10 of 22 cementless cups and 15 of 47 cemented cups were revised, primarily due to aseptic loosening. There was a difference in TT between revised and non-revised cups of 1.75 mm (CI 0.45–3.05) for cementless cups at 1-year follow-up, and of 1.11 mm (CI 0.30–1.93) for cemented cups at 2-year follow-up. Increased pain in activity and Disabilities of the Arm, Shoulder, and Hand (QDASH) score was associated with revision. At 10 years, patients with retained cups reported high satisfaction and grip strength, pain, and QDASH improvement above the minimal clinically important difference compared with preoperatively.

**Conclusion:**

10-year cup migration was similar between Motec cup groups. Revised cups migrated more than non-revised cups in both groups, and pain in activity and QDASH score were associated with revision. As such, decline in clinical outcomes, as well as progression in cup TT, seem to be useful predictors of cup revision.

The trapeziometacarpal (TMC) total joint arthroplasty is becoming an accepted standard in the treatment of symptomatic TMC arthritis. TMC total joint implants have undergone several design iterations since their introduction in the 1970s, yet have retained the core design principle of ball-in-socket articulation [1]. The Motec cup was produced in cementless and cemented versions with collars. The cementless Motec cup was abandoned in 2016 mainly due to high early revision rates [2]. The cemented Motec was marketed as a salvage cup for the cementless cup and was also discontinued in 2016 despite no published results. Many patients still have these implants in situ, but the long-term clinical and radiological outcomes are unknown.

Radiostereometric analysis (RSA) is a precise, validated radiographic modality to evaluate implant fixation and identify underperforming implant designs in upper and lower extremity implant designs [3-6]. The migration pattern of failed and non-failed implants may reveal interesting details on implant failure patterns. Few clinical studies have utilized RSA to assess TMC arthroplasty migration [3,5,7], long-term RSA studies are warranted, and the Motec cemented and cementless cups have never been investigated with RSA.

We aimed to evaluate the 10-year migration, clinical outcomes, survival, and revisions of cementless and cemented Motec cups. Additionally, early migration of revised and non-revised cups, and the association between clinical outcomes and cup revision, were investigated.

## Methods

### Study design

In a single-center prospective cohort study, we included patients undergoing total Motec TMC joint arhroplasty (Swemac, Linköping, Sweden) between 2009 and 2012 at Gødstrup Hospital, Herning, Denmark. From 2009 to 2010 a cementless Motec screw-and-collar TMC cup was used (cementless group), and from 2011 to 2012 a cemented all-polyethylene-collared TMC cup (cemented group) was used. The study was reported in accordance with the STROBE statement [8].

### Participants

Inclusion criteria were age between 18 and 75 years, symptomatic TMC osteoarthritis with Eaton-Glickel stage 2–4, and failed conservative treatment. Patients with symptomatic TMC osteoarthritis and the following criteria received a trapeziectomy: age at or above 75 years, severe scapho-trapezio-trapezoid (STT) arthritis, low functional demand, dementia, request for trapeziectomy, osteoporosis, alcohol or drug abuse, severe deformity of the trapezium or 1st metacarpal malunion. Other TMC arthroplasty brands were used during the Motec recruitment period.

### Components

**Figure 1:**
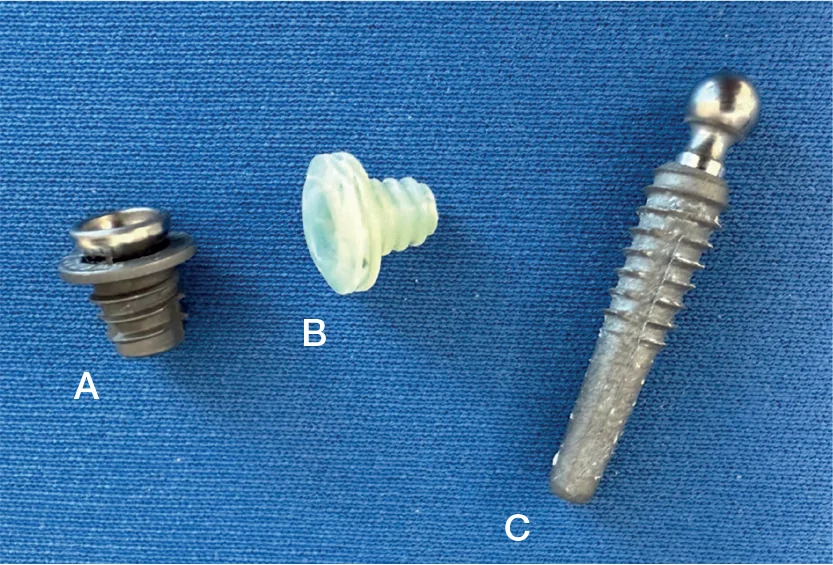
Motec TMC total joint prosthesis. (A) Cementless screw-cup assembled with a chrome-cobalt insert for articulation. (B) Cemented all-polyethylene cup. (C) Cementless screw-stem assembled with an Ø 6 mm head/neck component (the same stem and head/neck implants were used for both cups).

The cementless Motec TMC arthroplasty implant (Swemac, Linköping, Sweden) consisted of a screw-and-collar conical cup made of titanium alloy with a hydroxyapatite coating (Bonit, Swemac, Linköping, Sweden), and 2 surface components (cup and metacarpal head/neck inserts), which comprised a metal-on-metal (MoM) articulation (brom-nitrid treated chrome-cobalt-molybdenum alloy) (Figure 1A). The cemented Motec TMC arthroplasty implant (Swemac, Linköping, Sweden) consisted of an ultra-high-molecular-weight (UHMWPE) all-polyethylene-collared conical cup with parallel grooves. The polyethylene cup comprised a metal-on-polyethylene (MoP) articulation with the head/neck component (brom-nitrid treated chrome-cobalt-molybdenum alloy) (Figure 1B). The all-polyethylene cup was inserted with Palacos bone cement (Heraeus, Hanau, Germany). For both cup types, the diameter of the head of the head/neck segment was 6.0 mm and it was inserted into a cementless metacarpal screw stem of titanium alloy with a hydroxyapatite coating (Bonit, Swemac, Linköping, Sweden).

### Procedure

All patients were operated on by a senior surgeon (TBH). Procedures were performed under general anaesthesia using a tourniquet. Cefuroxime 1,500 mg intravenously was used as antibiotic prophylaxis. The TMC joint was exposed using a dorso-radial approach. After bone reaming, 5 tantalum beads (Ø 1.0 mm) were inserted into the trapezium bone widely spread in a predefined pattern using a bead gun (Kulkanon, Wennbergs Finmek, Gunnilse, Sweden), and the cementless cup and stem were screwed into the trapezium and the first metacarpal, respectively. The cemented polyethylene cups were fixed using open-mixed high-viscosity Palacos R+G Bone Cement (Heraeus, Hanau, Germany), which was pressurized into the reamed cavity using a blunt instrument before inserting the polyethylene cup. The polyethylene cup was prepared by a company (Wennbergs Finmek, Gunnilse, Sweden) with removal of the metal ring (intended for radiology visibility) and insertion of 4 tantalum beads (0.8 mm) in the rim and 1 at the cup dome.

### Radiostereometric analysis

**Figure 2:**
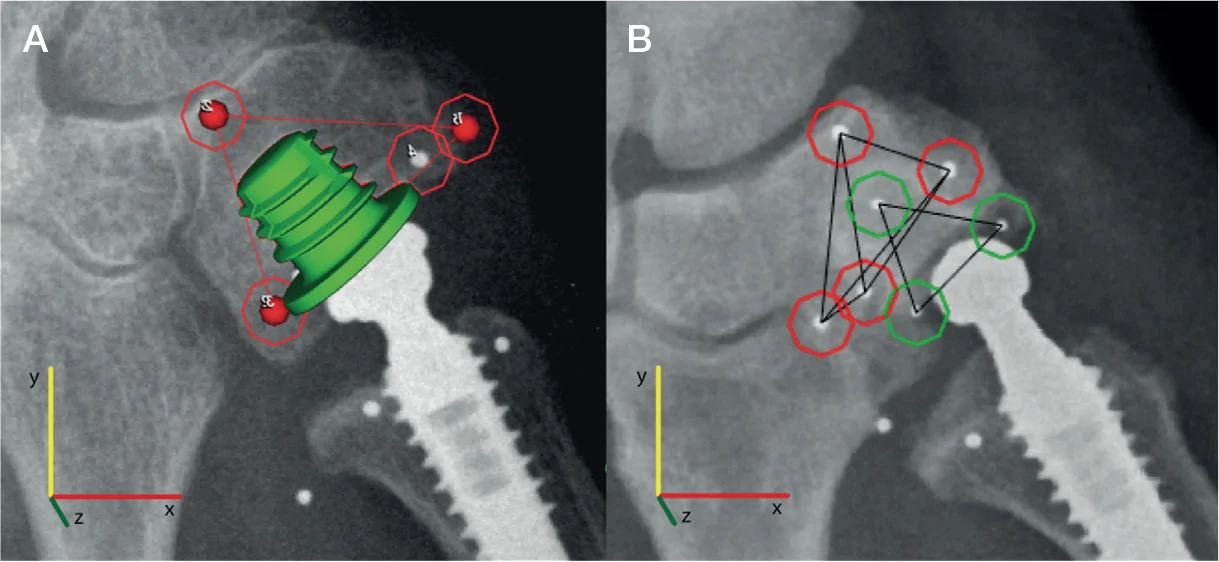
RSA analysis of (A) the cementless Motec cup (model-based RSA, green model) and (B) the cemented Motec all-polyethylene cup (marker-based RSA, green markers). The red markers are the bone markers inserted in the trapezium bone used as reference for the migration evaluation of the cup.

A standardized and previously described RSA setup was used [3]. RSA imaging was performed after cast removal (baseline = 3 weeks), at 3 months, and at 1, 2, 5, and 10-year follow-up. The 10-year follow-up window for patients with RSA was mean 119 months (SD 0.4) for the cementless group (n = 3) and mean 123 months (SD 4.5) for the cemented group (n = 10). All RSA images were analyzed by 1 observer, using the Model-Based RSA software (RSA*core*, Leiden, The Netherlands) (Figure 2). Model-based RSA was used for analysis within the calibration box coordinate system for both cup types. The upper limit for the mean error in rigid-body fitting of tantalum beads on the radiograph was 0.35 mm. Implant migration was evaluated with signed translations: x (+radial/–ulnar), y (+subsidence/–lift-off), z (+dorsal/–volar), and the summed total translation (TT) of the trapezium component, calculated by use of the Pythagorean theorem, TT = √(x^2^ + y^2^ + z^2^) [9].

### RSA precision

Double RSA examinations were obtained for 10 cementless and 28 cemented cups at 6-month or 1-year follow-up. The second RSA image was taken after repositioning of the RSA equipment [10]. The baseline RSA image served as the reference radiograph for analyzing the double RSA images. The bias was calculated as the difference in migration between the 2 double examinations, and precision was the standard deviation (SD) of the difference (Table S1, see Supplementary data). The mean condition number for the marker-models was 160 (SD 84) for the trapezium, and 236 (SD 86) for the polyethylene cup. The mean rigid-body error was 0.09 mm (SD 0.07) for trapezium bone markers and 0.07 mm (SD 0.08) for polyethylene cup markers. The model pose estimation error for cementless cups was mean 0.11 mm (SD 0.02 mm).

### Radiographic assessment

Radiographs from preoperative through the last available radiograph at cup failure/revision or 10-year follow-up were evaluated in consensus among 3 authors (ER, MS, JKT). The following were assessed: Eaton–Glickel osteoarthritis stage, preoperative presence and development of scapho-trapezio-trapezoid (STT) osteoarthrosis during follow-up, presence of postoperative radiolucent lines of minimum 0.5 mm, signs of aseptic cup-loosening and stem loosening, degree of heterotophic ossification, and dislocation.

### Clinical rehabilitation and examination

All operated on hands were immobilized in Plaster of Paris for 3 weeks postoperatively. Gradual increases in activity were allowed until 3 months postoperatively. Thereafter, patients resumed normal activity without restrictions. Patients were assessed clinically preoperatively, after 3 months, at 1, 2, 5, and 10 years following their surgery, or until the last follow-up before cup failure/revision or loss to follow-up. Patients who declined clinical follow-up were contacted by phone regarding cup revision and overall satisfaction. The clinical evaluation consisted of grip strength measurement (kg) (Baseline Digital Hand Dynamometer, Fabrication Enterprises Inc, White Plains, NY, USA), patient-reported outcomes (PROMs), pain at rest and in activity reported on a numeric rating scale (NRS 0 to 10) (0 = no pain, 10 = worst pain), and Disability of the Arm, Shoulder, and Hand score (QDASH 0 to 100) (0 = asymptomatic status, 100 = worst level of disability and symptoms). The minimal clinically important difference (MCID) for pain evaluation with the NRS score was 2 points [11,12] for QDASH 18 points [13], and for grip strength 0.84 kg [14].

The 10-year follow-up further included measurements of pinch strength (kg) (Baseline Electronic Pinch Gauge, Fabrication Enterprises Inc, White Plains, NY, USA), Kapandji score, and patient-reported satisfaction with the procedure and willingness to repeat surgery (NRS 0 to 10).

Revisions were defined as adverse events related to the arthroplasty that required cup revision due to aseptic loosening, septic loosening, traumatic dislocation, perioperative fracture leading to cup loosening, infections, trauma, or polyethylene wear. The cause for revision was prospectively registered in the database and validated by retrospective evaluation of radiographs and patient records.

### Sample size

Based on a previous RSA study comparing cementless (SD 0.16 mm) and cemented (SD 0.10 mm) TMC cup TT migration (mm) [3], each group should include a minimum of 17 implants to detect a difference in TT of 1 mm with a power set to 80% and an alpha of 5%. The cementless group consisted of 22 cups, and the cemented group of 47 cups.

### Statistics

RSA migration and secondary outcomes, including patient-reported outcomes (QDASH and pain scores) and clinical measures (grip strength and Kapandji score), were analyzed using linear mixed-effects models with follow-up time and implant type (or revision) as fixed effects and patient as a random effect. Models were estimated using maximum likelihood to include all available observations. For patients with bilateral procedures, dependency between implants was accounted for by including the patient as an additional random effect in the mixed-effects models. Group means over time were estimated from the mixed-effects models as model-based predicted values (least squares).

Missing data occurred due to technical limitations that prevented RSA analysis on certain follow-up occasions, and, in some cases, due to prosthesis failure leading to discontinuation of follow-up. To assess robustness of the findings with respect to missing data and analytical approach, sensitivity analyses including mixed-effects model, last observation carried forward, and 2-sample t-tests at each follow-up time point based on available data were performed (Table S2, see Supplementary data).

Survival rates were estimated using Kaplan–Meier analysis with revision of the cup component as the endpoint. Associations between continuous variables were assessed using Pearson’s correlation coefficient (rho), and logistic regression was used to estimate odds ratios for binary outcomes. Cup migration above 1 mm was evaluated as a threshold for revision risk [3]. Statistical analyses were performed using Stata (StataCorp, College Station, T, USA). A 2-sided P value < 0.05 was considered statistically significant.

### Ethics, registration, use of AI tools, funding, and disclosures

Before study initiation, the protocol was reviewed by the Central Denmark Region committee on Health Research Ethics, and rated as a quality assurance study, which, according to Danish law, was not reportable at the time (“Act on a Biomedical Research Ethics Committee System and the Processing of Biomedical Research Projects, Part 3”). The project was registered with the Danish Data Protection Agency (jr. nr.: 2007-58-0010), and the patients provided informed consent to participate in follow-up using RSA, which involved the insertion of markers into the polyethylene cup. The Helsinki II declaration was followed, and all data was handled according to the General Data Protection Regulation (GDPR). ChatGPT was used to optimize the flow and clarity of already written textual content. This study did not receive any grants or funding. The authors declare no conflict of interest. Complete disclosure of interest forms according to ICMJE are available on the article page, doi: 10.2340/17453674.2026.46544

## Results

**Figure 3:**
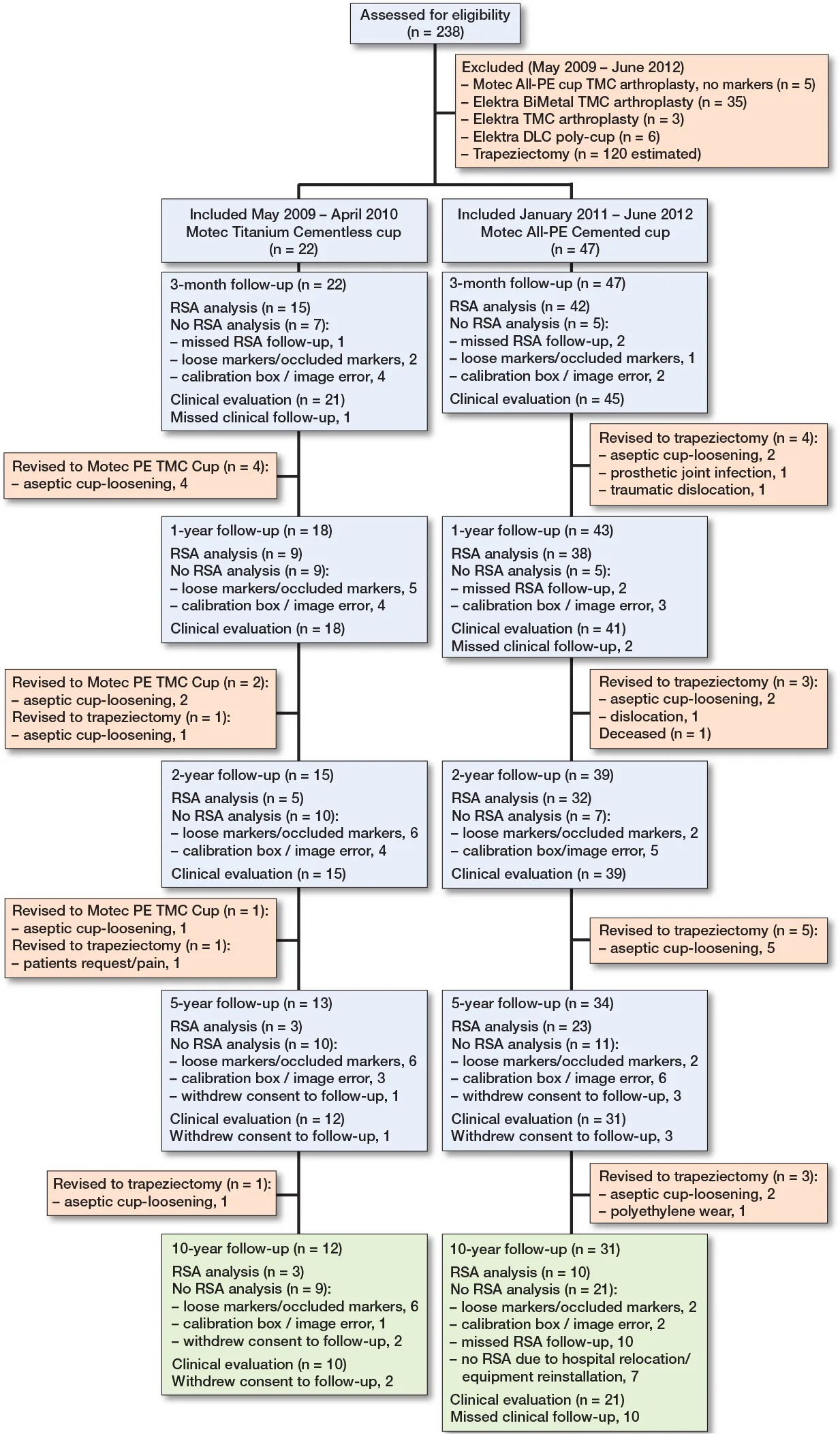
Flowchart up to 10 years of follow-up.

The cementless Motec screw cup was inserted in 21 patients (22 hands) at a mean age of 61 years (range 46–72), and the cemented Motec all-polyethylene cup was inserted in 41 patients (47 hands) at a mean age of 60 years (range 47–74) (Figure 3). 1 patient died during follow-up. Patients who declined to participate in follow-up reported being satisfied with their results. Demographics are given in Tables 1 and 2.

**Table 1:** Patient demographics by revision status

Factor	Revised cups	Non-revised cups	StdDiff
Number of hands	26	43	
Mean age (range)	60 (47–72)	60 (46–74)	0.01
Sex (male/female)	6/20	11/32	0.06
Side (right/left)	12/14	19/24	0.04

StdDiff: standardized difference.

**Table 2:** Patient demographics by fixation group

Factor	Cementless group	Cemented group	StdDiff
Number of hands	22	47	
Mean age (range)	61 (46–72)	60 (47–74)	0.10
Sex (male/female)	8/14	9/38	0.39
Side (right/left)	8/14	23/24	0.25
Cup size (7 mm/8.5 mm)	3/19	47 **^a^**	–
Neck size (small/medium/large **^b^**)	2/5/5	14/20/11	0.01
Eaton–Glickel grade (1/2/3/4)	0/4/16/2	0/12/27/8	0.23
Preoperative STT-joint arthritis, n	2	8	

StdDiff: standardized difference; STT: scapho-trapezio-trapezoid.

a Only available in 1 size (8.5 mm).

b Neck size not available from patient journals in 10 cementless and 2 cemented implants.

### Cup migration

At the 10-year follow-up, the mean difference in TT between groups was 0.54 mm (CI –0.68 to 1.77) higher in the cemented group. The migration pattern showed radial translation and lift-off in the cementless group, while radial–dorsal combined with subsidence in the cemented group (Table 3).

**Table 3:** Cup migration (mm) from baseline (3 weeks postoperatively) until 10 years follow-up (mixed model adjusted predictions)

Follow-up (months)	n	Cementless group Mean (CI)	n	Cemented group Mean (CI)	Difference (CI)	P value
x-translation (+ radial/– ulnar)						
3	15	0.04 (–0.25 to 0.33)	42	0.07 (–0.10 to 0.25)	–0.04 (–0.37 to 0.30)	0.8
12	9	0.48 (0.11 to 0.85)	38	0.30 (0.12 to 0.49)	0.17 (–0.2 to 0.58)	0.4
24	5	0.40 (–0.08 to 0.88)	32	0.30 (0.11 to 0.50)	0.10 (–0.42 to 0.62)	0.7
60	3	0.29 (–0.32 to 0.90)	23	0.21 (–0.01 to 0.44)	0.07 (–0.58 to 0.72)	0.8
120	3	0.32 (–0.29 to 0.93)	10	0.32 (–0.01 to 0.65)	0.00 (–0.69 to 0.69)	0.9
y-translation (+ subsidence/– lift-off)						
3	15	–0.09 (–0.48 to 0.30)	42	0.06 (–0.18 to 0.29)	–0.15 (–0.61 to 0.31)	0.5
12	9	–0.17 (–0.67 to 0.32)	38	0.42 (0.17 to 0.66)	–0.59 (–1.14 to –0.03)	0.04
24	5	0.18 (–0.46 to 0.83)	32	0.45 (0.19 to 0.72)	–0.27 (–0.97 to 0.42)	0.4
60	3	–0.13 (–0.94 to 0.68)	23	0.61 (0.30 to 0.91)	–0.74 (–1.60 to 0.13)	0.1
120	3	–0.11 (–0.91 to 0.70)	10	0.86 (0.45 to 1.32)	–0.99 (–1.9 to –0.07)	0.03
z-translation (+ dorsal/– volar)						
3	15	–0.12 (–0.50 to 0.26)	42	0.03 (–0.20 to 0.26)	–0.15 (–0.60 to 0.29)	0.5
12	9	0.07 (–0.41 to 0.55)	38	0.39 (0.15 to 0.62)	–0.32 (–0.85 to 0.22)	0.3
24	5	0.15 (–0.47 to 0.77)	32	0.50 (0.24 to 0.75)	–0.35 (–1.02 to 0.32)	0.3
60	3	0.01 (–0.75 to 0.78)	23	0.29 (0.00 to 0.58)	–0.28 (–1.1 to 0.55)	0.5
120	3	–0.01 (–0.78 to 0.76)	10	0.52 (0.10 to 0.94)	–0.53 (–1.40 to 0.35)	0.2
Total translation (TT)						
3	15	0.36 (SE 0.28)	42	0.22 (SE 0.17)	0.14 (–0.49 to 0.77)	0.7
12	9	1.11 (SE 0.34)	38	0.85 (SE 0.17)	0.26 (–0.50 to 1.01)	0.5
24	5	0.98 (SE 0.44)	32	1.04 (SE 0.18)	–0.06 (–0.10 to 0.88)	0.9
60	3	0.69 (SE 0.55)	23	1.31 (SE 0.21)	–0.62 (–1.78 to 0.53)	0.3
120	3	0.79 (SE 0.55)	10	1.33 (SE 0.30)	–0.54 (–1.77 to 0.68)	0.4

CI: 95% confidence interval.

SE: standard error.

At 1-year follow-up, there was RSA follow-up on 4 revised and 5 non-revised cups in the cementless group, and on 11 revised and 27 non-revised cups in the cemented group (Table S3, see Supplementary data). The 1-year mean TT was higher for revised cementless cups (2.07 mm, SE 0.49) than for non-revised cementless cups (0.32 mm, SE 0.45; P = 0.008). There was no difference in mean TT of revised cemented cups (1.18 mm, SE 0.30) compared with non-revised cemented cups (0.71 mm, SE 0.20; P = 0.2). However, the mean 2-year TT for revised cemented cups (1.90 mm, SE 0.36) was higher than for non-revised cemented cups (0.78 mm, SE 0.20; P = 0.007).

**Figure 4:**
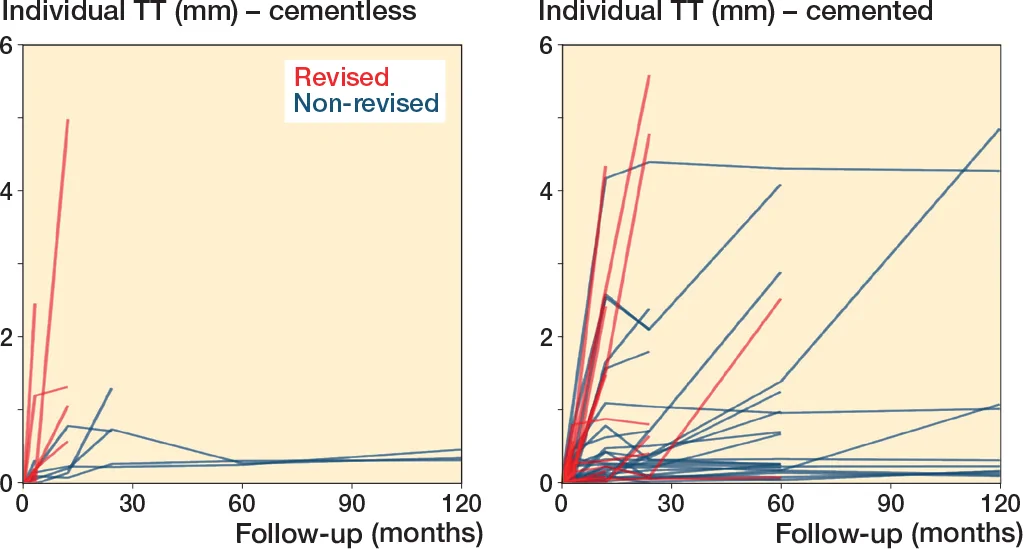
Individual line plots of the cementless and cemented cup total translation during the 10-year follow-up. One cementless cup with failure (90° cup rotation on radiographs) is displayed in the revised group (total translation of 5 mm).

Cup migration > 1 mm TT was observed in 22 of 59 cups with analyzable RSA images at the last follow-up (n = 5 cementless, n = 17 cemented). 3 cementless and 5 cemented cups with cup migration > 1 mm were revised during the 10-year follow-up (Figure 4, Table 4). For all cups combined, there was no clinically relevant association between TT and QDASH score (rho = 0.02, P = 0.9), or between TT and pain during activity (rho = –0.15, P = 0.3), at the patient’s last available RSA follow-up.

**Table 4:** Revised cups: data on total translation, pain at rest, and in activity is reported at the last available follow-up prior to cup revision

Group Sex	Side	Months to revision	Months RSA follow-up	TT (mm)	NRS pain		Cause of revision	Revised to
					at rest	in activity		
Cementless group								
Female	Right	3	3	2.48	3	6	Aseptic cup-loosening	CC
Male	Left	6	3	0.09	0	0	Aseptic cup-loosening	CC
Male	Right	7	3	0.15	0	7	Aseptic cup-loosening	CC
Male	Left	8	3	0.18	0	8	Aseptic cup-loosening	CC
Female	Left	13	12	0.59	4	5	Aseptic cup-loosening	CC
Female	Left	19	12	1.08	1	6	Aseptic cup-loosening	TE
Female	Right	22	3	0.18	0	0	Aseptic cup-loosening	CC
Male	Right	26	N/A	N/A	0	0	Aseptic cup-loosening	CC
Female	Left	40	12	1.34	0	0	Personal request	TE
Female	Left	60	N/A	N/A	2	6	Aseptic cup-loosening	TE
Cemented group								
Female	Right	5	3	0.36	0	7	Traumatic dislocation	TE
Female	Right	6	3	0.60	0	6	Aseptic cup-loosening	TE
Female	Left	7	3	0.14	2	7	Prosthetic joint infection	TE
Male	Left	11	3	0.14	4	8	Aseptic cup-loosening	TE
Female	Left	12	12	0.18	0	0	Dislocation	TE
Female	Right	12	12	2.44	4	6	Aseptic cup-loosening	TE
Female	Right	23	12	1.50	0	3	Aseptic cup-loosening	TE
Female	Left	24	12	4.36	3	3	Aseptic cup-loosening	TE
Female	Left	28	24	0.67	2	3	Aseptic cup-loosening	TE
Male	Right	50	24	4.80	0	0	Aseptic cup-loosening	TE
Female	Left	52	12	0.11	0	0	Aseptic cup-loosening	TE
Female	Right	57	60	2.54	0	0	Aseptic cup-loosening	TE
Female	Left	62	24	0.82	1	8	Aseptic cup-loosening	TE
Female	Left	64	24	0.43	2	10	Polyethylene wear	TE
Female	Right	71	60	0.10	0	3	Aseptic cup-loosening	TE

CC: Cemented cup; NRS: Pain reported on numeric rating scale; N/A: not applicable; TE: Trapeziectomy; TT: Total translation.

### Radiographic findings

Radiographic changes presented earlier for cementless cups than cemented cups regarding aseptic loosening (difference 4.2 months, CI –22.4 to 30.7) and radiolucent lines (difference 12.4 months, CI –10.1 to 34.8). Radiolucent lines of minimum 0.5 mm width on radiographs preceded radiographic aseptic cup-loosening. At the last available follow-up, radiolucent lines were more frequent than aseptic cup-loosening. No radiographically evident stem loosening was observed (Table 5). STT arthritis developed in 3 hands with cementless cups and 4 hands with cemented cups during the 10-year follow-up period. About half of the patients developed periarticular heterotophic ossifications. Dislocations occurred in 8 of 69 implants.

**Table 5:** Radiographic findings. Values are counts or as specified

Outcome	Cementless group	Cemented group	P value
Number of patients	22	47	
Mean time in months (CI) to			
radiolucent lines > 0.5 mm	25.5 (7.7–43.3)	37.9 (24.9–51.8)	0.8
aseptic cup loosening	36.3 (9.6–62.9)	40.4 (25.7–55.1)	0.3
At last follow-up			
Aseptic cup-loosening	12	19	0.3
Radiolucent lines > 0.5 mm	14	27	0.6
Heterotopic ossification with < 3 mm distance	7	24	0.1
Dislocation	1	7	0.2
STT-joint arthritis	5	12	0.8
Revision (trapeziectomy/Motec all-poly cup)	3/7	15/0	0.3

STT: scapho-trapezio-trapezoid, CI: 95% confidence interval.

### Clinical results

Overall, clinical results were similar between groups at all follow-up times (Table 6). Grip strength improved over time in both groups. At 10 years, grip strength was comparable to the preoperative strength of the patients’ contralateral hand. Further, no clinically relevant difference in grip strength (1 kg, CI –6 to 8) and pinch strength (0.4 kg, CI –0.6 to 1.4) was observed between groups at 10-year follow-up (Table 6).

**Table 6:** Patient-reported outcomes and clinical outcomes throughout the 10 years of follow-up

Follow-up/Outcome	Cementless group	Cemented group	Difference (CI)	P value
Preoperative, n	21	41		
Grip strength, mean kg (CI) **^a^**	22 (17–27)	21 (18–25)	1 (–6 to 7)	0.9
Contralateral hand	29 (24–34)	27 (23–31)	2 (–4 to 8)	0.6
Pain at rest, mean NRS (SE)	2.7 (0.3)	3.4 (0.2)	–0.6 (–1.5 to 0.2)	0.1
Pain in activity, mean NRS (SE)	7.6 (0.5)	8.0 (0.4)	–0.4 (–1.7 to 0.8)	0.5
QDASH score, mean (CI)	34 (26–41)	39 (34–44)	–5 (–14 to 4)	0.2
3-month follow-up (n)	21	45		
Grip strength, mean kg (CI) **^a^**	21 (16–26)	20 (17–24)	1 (–5 to 7)	0.8
Pain at rest, mean NRS (SE)	0.5 (0.3)	0.5 (0.2)	0.0 (–0.8 to 0.8)	0.9
Pain in activity, mean NRS (SE)	2.8 (0.5)	3.4 (0.4)	–0.6 (–1.9 to 0.6)	0.3
QDASH score, mean (CI)	20 (12–27)	25 (20–30)	–5 (–14 to 4)	0.3
1-year follow-up (n)	18	41		
Grip strength, mean kg (CI) **^a^**	27 (21–32)	24 (21–28)	2 (–4 to 8)	0.7
Pain at rest, mean NRS (SE)	0.4 (0.4)	0.9 (0.2)	–0.4 (–1.3 to 0.4)	0.3
Pain in activity, mean NRS (SE)	2.2 (0.5)	2.5 (0.4)	–0.3 (–1.6 to 1.0)	0.7
QDASH score, mean (CI)	14 (6–21)	17 (12–22)	–4 (–13 to 6)	0.4
2–year follow-up (n)	15	39		
Grip strength, mean kg (CI) **^a^**	28 (23–33)	27 (23–31)	1 (–6 to 7)	0.8
Pain at rest, mean NRS (SE)	0.1 (0.4)	0.6 (0.3)	–0.5 (–1.4 to 0.4)	0.3
Pain in activity, mean NRS (SE)	0.7 (0.6)	2.3 (0.4)	–1.6 (–3.0 to –0.3)	0.02
QDASH score, mean (CI)	12 (4–20)	15 (10–21)	–3 (–13 to 6)	0.5
5-year follow-up (n)	12	31		
Grip strength, mean kg (CI) **^a^**	25 (20–31)	27 (23–31)	–2 (–8 to 5)	0.6
Pain at rest, mean NRS (SE)	0.3 (0.4)	0.5 (0.3)	–0.2 (–1.2 to 0.86)	0.8
Pain in activity, mean NRS (SE)	1.4 (0.7)	2.4 (0.4)	–1.0 (–2.5 to 0.5)	0.2
QDASH score, mean (CI)	16 (10–21)	14 (9 –20)	2 (–8 to 12)	0.7
10-year follow-up (n)	10	21		
Grip strength, mean kg (CI) **^a^**	25 (20–31)	25 (20–29)	1 (–6 to 8)	0.8
Pinch strength, mean kg (CI) **^a^**	3.8 (3.0–4.5)	3.3 (2.7–3.9)	0.4 (–0.6 to 1.4)	0.4
Contralateral hand	3.8 (3.0–4.5)	3.3 (2.7–3.9)	0.4 (–0.6 to 1.4)	0.4
Pain at rest, mean NRS (SE)	0.3 (0.5)	0.6 (0.3)	–0.4 (–1.5 to 0.7)	0.5
Pain in activity, mean NRS (SE)	0.6 (0.7)	1.7 (0.5)	–1.1 (–2.8 to 0.6)	0.2
QDASH score, mean (CI)	13 (4–21)	16 (10–22)	–3 (–14 to 8)	0.6
Kapandji score, median (IQR)	10 (9–10)	10 (9–10)	–	0.8
Willingness to repeat, median (IQR)	10 (10–10)	10 (9–10)	–	0.3
Satisfaction, median (IQR)	10 (10–10)	10 (10–10)	–	0.3

IQR: interquartile range; CI: 95% confidence interval; SE: standard error; QDASH: Disabilities of the Arm, Shoulder, and Hand (range 0–100, 100 best outcome), NRS: numeric rating scale (range 0–10, 10 best outcome).

a Operated on hand.

Pain at rest was clinically relevant and statistically significantly reduced at 3 months’ follow-up and the pain level remained below mean 1 NRS in both groups until 10-year follow-up. Likewise, pain in activity was reduced in both groups compared with the preoperative pain. Pain in activity at the last available follow-up were associated with cup revision. For each 1-unit NRS increase in pain activity score, the odds of revision increased by 48% (OR = 1.48, CI 1.20–1.82).

The QDASH score improved from preoperative throughout the 10-year follow-up by a mean of 21 points (CI 13–29) in the cementless group, and by a mean of 23 points (CI 18–29) in the cemented group (P < 0.001). The 10-year difference in QDASH score was a mean of 3 points (CI –8 to 14) between groups (P = 0.6). QDASH score at the last available follow-up was associated with cup revision. For each 1-point increase in QDASH score, the odds of revision increased by 5% (OR = 1.05, CI 1.02–1.08).

The 10-year satisfaction with the surgical outcome and willingness to repeat the procedure were excellent in patients with retained implants (Table 6).

### Revisions and cup survival

**Figure 5:**
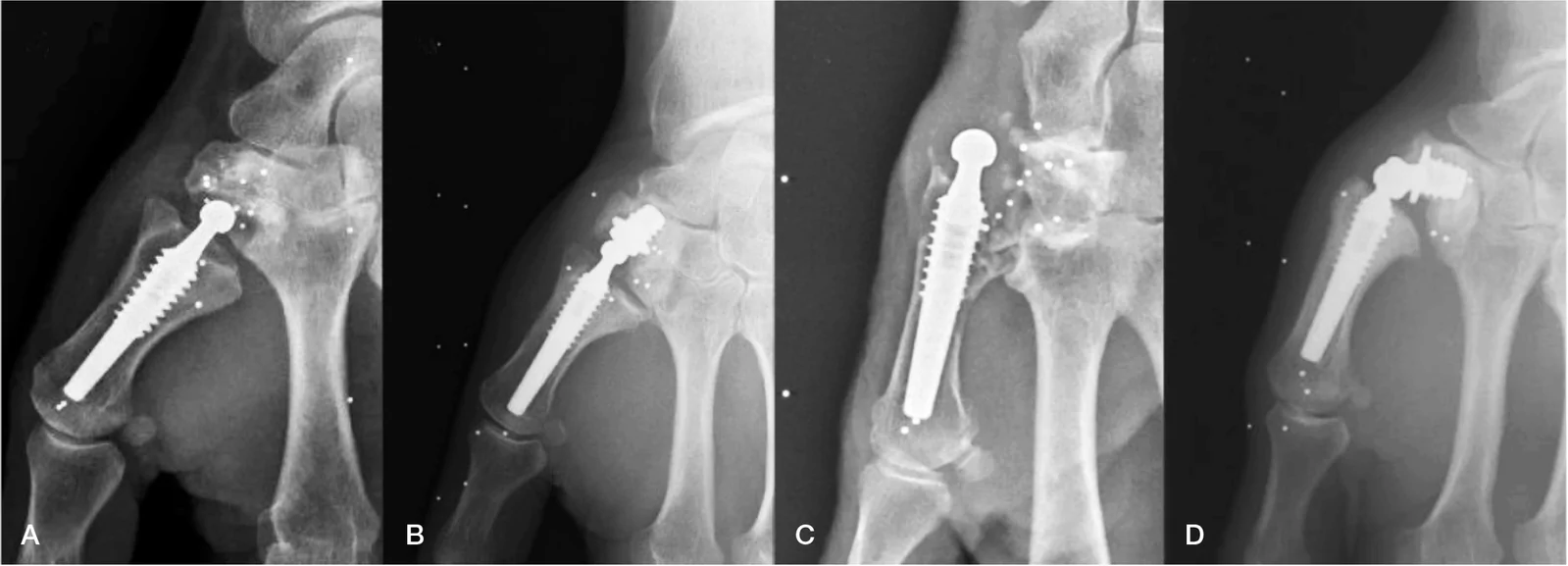
Radiographic implant failures with (A) osteolysis around the proximal stem due to pseudotumor/wear debris, (B) cementless cup with subsidence to the bottom of the trapezium and impingement between the 1st metacarpal and trapezium leading to disconnection of the articulation, (C) dislocation of the head from an all-polyethylene cup, and (D) aseptic cup-loosening, leading to cup tilt in the trapezium bone.

In the cementless group, 10 of 22 cups were revised, and another cup presented with radiological failure but was not revised. In the cemented group, 15 of 47 cups were revised during the 10-year follow-up, leading to a risk difference of revision of 18% (CI –7 to 43). 4 of the 8 patients with bilateral TMC joint arthroplasty underwent revision, but no bilateral revisions were performed. There was no difference in patient age, sex, and operated side between non-revised and revised cups. All cemented cups were revised with a trapeziectomy with preservation of the stem in situ, while 7 of the 10 revised cementless cups were converted to cemented all-polyethylene cups. Aseptic cup-loosening was the most frequent reason for cup revision, accounting for 9 of 10 and 12 of 15 of all revisions in the cementless and cemented groups, respectively, leading to a risk difference for aseptic loosening of 10% (CI –17 to 37) (see Table 4, Figure 5).

**Figure 6:**
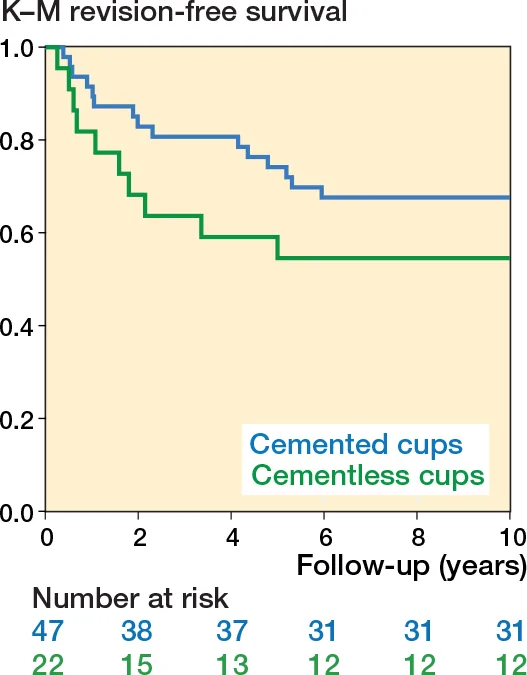
Kaplan–Meier survival plot of cementless and cemented Motec cups.

The estimated 10-year Kaplan–Meier cup survival was 55% (CI 32–72) for the cementless group and 68% (CI 52–79) for the cemented group (P = 0.2) (Figure 6). The risk of revision was 65% higher in the cementless group compared with the cemented group (HR 1.65, CI 0.74–3.68), P = 0.2).

## Discussion

We aimed to evaluate 10-year migration, clinical outcomes, and revisions of cementless and cemented Motec cups. We found that, during 10 years of follow-up, cup migration was similar in the cementless and the cemented Motec cups. The early revision rate was high, primarily due to aseptic loosening. Revised cups migrated more than non-revised cups in both groups at early follow-ups, and activity-related pain and QDASH score were associated with revision.

Knowledge of trapeziometacarpal cup fixation is limited, with only 2 clinical studies on ball-and-socket implants published to date [3,7]. The reported cup TT was 0.19 mm for the cementless Elektra conical screw-cup and 0.24 mm for the cemented spherical all-polyethylene DLC cup at 2-year follow-up [3], and 0.39 mm for the cementless Moovis conical press-fit cup at 5-year follow-up [7]. The TT of both the cementless and cemented Motec cups in the present study was higher from 1-year up to 10-year follow-up. For trapeziometacarpal cups no acceptance threshold for migration has been established. However, an association between cup TT above 1 mm and aseptic cup-loosening leading to revision (n = 2) has been cautiously suggested as a clinically relevant cup migration limit [3]. In experimental studies of TMC cup fixation, a more conservative TT limit of 0.5 mm for cup failure has been utilized [15,16].

In the current study, early migration was higher in revised vs non-revised cups for both the cementless group (1 year) and the cemented group (2 years). Approximately 50% of revised cementless and cemented Motec cups had more than 0.5 mm TT at the last available follow-up before cup revision. The cup migration pattern of Elektra and DLC cups, which had no collar, was subsidence into the trapezium [3]. Contrarily, both the cementless and cemented Motec cups in the present study had a collar design to resist cup subsidence, and this migration pattern was confirmed by RSA measurements for cementless Motec cups, while the cemented Motec cups subsided with the cement mantle. The collar design also introduces a rotation center outside of the trapezium, which increases the stress on the Motec cup during angular loading, such as thumb pinch and grip [2,17]. Accordingly, both cementless and cemented Motec cups exhibited radial tilt during follow-up. The rotation center is more eccentric in the cementless Motec cup design, due to the cup insert, which may induce a higher load on the fixation interface with risk of early cup micromotion and compromised osseointegration. This likely explains the early loosening and revision of cementless Motec cups especially. For cemented cups, progressive subsidence, and radial and dorsal cup tilt, implied a gradual loss of support, consistent with the slightly later cup loosening and time to revision in this group. Radiographic evaluation of the cement mantle and all-polyethylene cup was difficult but, based on the revision surgery description, aseptic loosening occurred in the cement–bone interface.

The clinical results of the cemented and cementless Motec arthroplasties align with other publications on trapeziometacarpal arthroplasties in terms of early and lasting pain relief, improved QDASH score, and grip strength above the MCIDs [7,18,19]. Additionally, the patients with Motec trapeziometacarpal arthroplasties in situ at 10 years reported excellent satisfaction and high willingness to repeat the procedure, similar to long-term clinical results with newer trapeziometacarpal arthroplasties [7,20]. Importantly, pain and high QDASH score at the final available follow-up were associated with Motec cup revision.

The 10-year survival rate of 55% for cementless Motec cups aligns with a previously reported 4-year survival rate of 50% [2]. Other cementless conical cup designs report better survival rates: 69% for the cementless Elektra screw-cup without a collar [21], and 98–100% at midterm for the cementless press-fit Moovis cup [7,22]. Likewise, the 10-year survival rate of 68% for cemented Motec cups is inferior to the 26-year survival rate of 74% for the cemented DLC spherical all-polyethylene cup [23]. During revision surgery, most cementless Motec cups could be salvaged with a cemented Motec cup, whereas cemented cups were converted to trapeziectomy [24].

### Strengths

Major strengths for this cohort study include a similar design for the 2 investigated TMC arthroplasties (collared and threads/grooves), which differ mainly by fixation method and cup material. All surgeries were performed by a single very experienced surgeon, which alleviates variability. RSA was conducted according to standard guidelines, ensuring comparability with other studies [10]. Condition numbers were high, but this is a known and acceptable limitation of small bones, and precision values were acceptable.

### Limitations

Marker occlusion, marker loosening, and image calibration and recording issues caused the RSA data to be incomplete for several hands. This may be solved in future studies using automated digitally reconstructed radiographs and CT volumetric bone models for evaluation [25]. Due to the high precision and accuracy of RSA, small patient cohorts are generally sufficient to study the effect on prosthetic fixation including for implants in the upper extremity [26]. However, due to the small number of patients available for the 10-year follow-up, the risk of type II error cannot be excluded. Patient hand dominance could potentially affect the hand load and cup migration, but data on hand dominance was not available in the current study. However, previous studies indicate that manual labor and more strenuous work do not lead to increased revision rates [27].

### Conclusion

10-year cup migration was similar between Motec cup groups. Revised cups migrated more than non-revised cups in both groups at early follow-up, and pain in activity and QDASH score were associated with cup revision. In perspective, a decline in clinical outcomes, as well as progressive cup TT, seem to be useful predictors of cup revision.

Although newer TMC arthroplasty implant designs demonstrate improved implant survival and retain the possibility of conversion to trapeziectomy, their increasing use in younger, higher-demand patient populations presents new challenges. Notably, this trend highlights the need to develop standardized, universally compatible revision systems. Such systems must accommodate the heterogeneity of existing implant designs, particularly variations in stem-trunnion interfaces and head sizes of the head/ neck components.

### Supplementary data

Supplementary Tables S1–S3 are available as supplementary data on the article page, doi: 10.2340/17453674.2026.46544

## Supplementary Material



## Notes

ER contributed to research design, data acquisition, data interpretation and writing the first draft of the manuscript. TBH contributed to research design and data acquisition. MS contributed to research design, data acquisition, RSA analysis, data interpretation, and writing the first draft of the manuscript. JKT contributed to research design, data acquisition, performed the data analysis, data interpretation, and writing the first draft of the manuscript. All authors revised and approved the final version of the manuscript.

The authors would like to thank project nurse Anne-Dorthe Riedel for her valued help with project coordination and RSA analyses.

Handling co-editors: Bart Pijls and Robin Christensen

*Acta* thanks Gerald A Kraan, Elise Laende and Marco van der Pluijm for help with peer review of this manuscript.
