# Supplementary material for: Implant migration and clinical outcomes with cemented vs cementless Motec cups in total trapeziometacarpal arthroplasty: a 10-year prospective RSA cohort study of 69 patients
Source: Acta Orthop. 2026 Sep 9;97:642–51. doi: 10.2340/17453674.2026.46544 (PMC13555598; doi:10.2340/17453674.2026.46544)
Supplement: Supplementary file 1 [file ActaO-97-46544-s1.pdf]

SUPPLEMENTARY DATA

Supplementary Table S1. RSA precision based on RSA double-examinations at 6-month or 1-year follow-up.

	Cementless group				Cemented group			
	x	y	z	TT	x	y	z	TT
Double examinations, n	10				21			
Mean difference [a]	0.05	0.03	–0.06	0.04	0.46	0.02	0.01	0.04
Precision (SD) [b]	0.09	0.11	0.35	0.26	0.29	0.10	0.24	0.14
Min. difference	–0.07	–0.07	–0.70	–0.23	–0.21	–0.06	–0.86	–0.15
Max. difference	0.25	0.32	0.33	0.67	1.50	0.49	0.76	0.70

[a] Mean difference: systemic error between two RSA double measurements (bias).
[b] Precision: reflects the lower limit within which it is possible to detect cup migration on an individual basis.
TT:Total translation

Supplementary Table S2. Sensitivity analyses of between-group differences in total translation.

**Group**	**Cementless group**			**Cemented group**				
Follow-up (Months)	n	Mean	SE	n	Mean	SE	Difference (CI)	P value
t test								
3	15	0.37	0.17	42	0.23	0.04	0.14 (–0.10 to 0.37)	0.2
12	9	1.06	0.51	38	0.81	0.18	0.25 (–0.64 to 1.14)	0.6
24	5	0.67	0.20	32	1.00	0.26	–0.33 (–1.67 to –1.01)	0.6
60	3	0.30	0.02	23	1.14	0.29	–0.85 (–2.54 to 0.84)	0.3
120	3	0.39	0.05	10	1.24	0.57	–0.85 (3.21 to –1.51)	0.4
Last available carried forward								
3	15	0.36	0.17	42	0.22	0.04	0.14 (–0.10 to 0.37)	0.2
12	16	0.82	0.32	43	0.75	0.16	0.06 (–0.60 to 0.72)	0.8
24	16	0.93	0.31	43	0.98	0.21	–0.05 (–0.85 to 0.75)	0.9
60	16	0.91	0.32	43	1.30	0.23	–0.39 (–1.25 to 0.47)	0.4
120	16	0.93	0.31	43	1.32	0.24	–0.39 (–1.27 to 0.49)	0.4
Mixed-model								
3	15	0.36	0.28	42	0.22	0.17	0.14 (–0.49 to 0.77)	0.7
12	9	1.11	0.34	38	0.85	0.17	0.26 (–0.50 to 1.01)	0.5
24	5	0.98	0.44	32	1.04	0.18	–0.06 (–0.10 to 0.88)	0.9
60	3	0.69	0.55	23	1.31	0.21	–0.62 (–1.78 to 0.53)	0.3
120	3	0.79	0.55	10	1.33	0.30	–0.54 (–1.77 to 0.68)	0.4

SE: Standard Error

CI: 95% Confidence Interval

Supplementary Table S3. Table of total translation in patients with non-revised cups compared with patients with revised implants or failed implants (mixed model adjusted predictions).

**Group**		**Revised cups**			**Non-revised cups**			
Follow-up (Months)	n	Mean	SE	n	Mean	SE	Difference (CI)	P value
Cementless group								
3	8	0.57	0.36	7	0.17	0.38	0.40 (–0.64 to 1.43)	0.5
12	4	2.07	0.49	5	0.32	0.45	1.75 (0.45 to 3.05)	**0.008**
24	0	–	–	5	0.68	0.45	–	
60	0	–	–	3	0.40	0.55	–	
120	0	–	–	3	0.49	0.55	–	
Cemented group								
3	16	0.26	0.26	26	0.20	0.20	0.06 (–0.58 to 0.70)	0.9
12	11	1.18	0.30	27	0.71	0.20	0.47 (–0.23 to 1.18)	0.2
24	7	1.90	0.36	25	0.78	0.20	1.11 (0.30 to 1.93)	**0.007**
60	2	2.01	0.63	21	1.18	0.22	0.83 (–0.48 to 2.14)	0.2
120	0	–	–	10	1.23	0.29	–	
Combined								
3	24	0.36	0.21	33	0.19	0.18	0.17 (–0.38 to 0.72)	0.5
12	15	1.43	0.26	32	0.65	0.18	0.78 (0.16 to 1.41)	**0.01**
24	7	1.99	0.36	30	0.76	0.19	1.23 (0.43 to 2.02)	**0.003**
60	2	2.12	0.63	24	1.08	0.20	1.03 (–0.27 to 2.35)	0.1
120	0	–	–	13	1.08	0.26	–	

SE: Standard Error

CI: 95% Confidence Interval
